# Comprehensive Review of Depression, Anxiety, and Insomnia in Oncologic Populations

**DOI:** 10.1192/j.eurpsy.2026.10661

**Published:** 2026-07-31

**Authors:** A. H. I. Abu Shehab, Ș. L. Burlea, A. Lucreția, I. Chiscop, A. A. Ș. Baltă, A. Bulgaru Iliescu, L. Stoica, A. Ciubară

**Affiliations:** 1 Psychiatry, “Elisabeta Doamna” Psychiatry Hospital, Galați, Galati; 2 Psychiatry, “Grigore T. Popa” University of Medicine and Pharmacy (UMF), Iași, Iasi; 3 Internal Medicine; 4 Oral & Maxillofacial Surgery, “Sf. Apostol Andrei” Emergency Clinical County Hospital, Galați; Faculty of Medicine & Pharmacy, “Dunărea de Jos” University of Galați; 5 Medical/Clinical Department & Doctoral School of Biomedical Sciences, Faculty of Medicine and Pharmacy, “Dunărea de Jos” University of Galați, Galati; 6 Morphofunctional Sciences, County Emergency Clinical Hospital “St. Spiridon” Iași; “Grigore T. Popa” University of Medicine and Pharmacy (UMF), Iași, Iasi; 7 Psycho-oncology, Psycho-oncology Clinic Lidia Stoica, Bucharest; 8 Psychiatry, Faculty of Medicine and Pharmacy, “Dunărea de Jos” University of Galați; “Elisabeta Doamna” Psychiatry Hospital, Galați, Galati, Romania

## Abstract

**Introduction:**

Depression, anxiety, and insomnia represent the most prevalent psychiatric comorbidities in cancer populations, significantly impacting quality of life, treatment adherence, and survival outcomes. These conditions are increasingly recognized as complex neurobiological phenomena rather than simple psychological reactions to cancer diagnosis.

**Objectives:**

To synthesize current evidence on the epidemiology, pathophysiology, assessment approaches, and treatment interventions for depression, anxiety, and insomnia in oncologic populations, providing evidence-based insights for clinical practice and research.

**Methods:**

Comprehensive review of recent meta-analyses, systematic reviews, and clinical guidelines published between 2023-2025. Key databases included PubMed, EMBASE, and major oncology journals. Priority was given to large-scale meta-analyses and evidence-based clinical practice guidelines from major oncology organizations.

**Results:**

Global meta-analysis of 135,015 cancer survivors from 31 countries revealed prevalence rates of 23.7% for depression, 24.4% for anxiety, and 34.1% for sleep disorders. Pathophysiological mechanisms involve immune-inflammatory processes, hypothalamic-pituitary-adrenal axis dysregulation, and neurotransmitter alterations. Validated screening tools include Hospital Anxiety and Depression Scale, Patient Health Questionnaire-9, Generalized Anxiety Disorder-7, and Insomnia Severity Index. Evidence-based treatments demonstrate strong efficacy: Cognitive Behavioral Therapy shows moderate to large treatment benefits for depression and anxiety, while Cognitive Behavioral Therapy for Insomnia shows very large improvements in sleep quality and duration. Mindfulness-based interventions receive strong American Society of Clinical Oncology recommendations. Pharmacological approaches favor selective serotonin reuptake inhibitors as first-line agents.

**Conclusions:**

Psychiatric comorbidities in cancer require systematic screening and evidence-based treatment. The high prevalence rates underscore the need for integrated psycho-oncology care. Future directions include personalized medicine approaches and prevention-focused interventions targeting inflammatory pathways.

**Disclosure of Interest:**

None Declared

